# Existential and Spiritual Attitudes of Polish Medical and Nursing Staff towards Death

**DOI:** 10.3390/ijerph19159461

**Published:** 2022-08-02

**Authors:** Krzysztof Zdziarski, Paulina Zabielska, Sylwia Wieder-Huszla, Iwona Bąk, Katarzyna Cheba, Mariola Głowacka, Beata Karakiewicz

**Affiliations:** 1 Subdepartment of Social Medicine and Public Health, Department of Social Medicine, Pomeranian Medical University in Szczecin, Żołnierska 48, 71-210 Szczecin, Poland; krzysztof.zdziarski@pum.edu.pl (K.Z.); beata.karakiewicz@pum.edu.pl (B.K.); 2 Department of Clinical Nursing, Pomeranian Medical University in Szczecin, Żołnierska 48, 71-210 Szczecin, Poland; sylwia.wieder.huszla@pum.edu.pl; 3 Department of Applied Mathematics in Economy, Faculty of Economics, West Pomeranian University of Technology in Szczecin, Piastów 17, 70-310 Szczecin, Poland; iwona.bak@zut.edu.pl (I.B.); katarzyna.cheba@zut.edu.pl (K.C.); 4 Department of Health Sciences, Masovian State University in Płock, Dąbrowskiego 2, 09-402 Płock, Poland; mariola.glowacka@wp.pl

**Keywords:** axiology, interaction, end of life, patient, medical and nursing staff

## Abstract

Issues related to death are difficult areas of human existence and are most often considered in terms of ethical or non-ethical behaviour. The aim of the research was to examine the attitudes of Polish medical and nursing personnel towards death. The research was conducted among medical (110 people) and nursing staff (110 people) working in Polish hospitals and representing 16 regions. The Questionnaire About Attitudes to Death (DAP-R-PL) was used. The attitude of medical staff, taking into account the holistic approach to the patient in his existential–spiritual dimension, is an extremely important element of professional care. The study outlines the attitudes of medical and nursing staff towards death. Medical personnel under examination demonstrate a mature attitude towards death. In light of this research, health care workers show great commitment to helping dying people with existential and spiritual needs.

## 1. Introduction

Death can be understood as a phenomenon, condition, process or experience with an associated negative, neutral or positive emotional attitude. Searching for a synonym for this word, we meet the following terms: end, end of life, descent, dying. Most of these concepts are pejorative, which confirms the opinion that man has a constant fear of death. According to psychologists, thanatical anxiety is most emotionally embedded in the human psyche and has the strongest potential for impact. Scientists are trying to systematize attitudes towards dying in order to provide existential calm, e.g., in the interaction of medical and nursing personnel. A review of the literature on the subject shows that there are different theories describing the problem of death. One of them talks about this approach to the dying person and emphasizes that the patient should be given maximum care, but at the same time treated as an object that can be freely manipulated. Another indicates a more ethical personalistic attitude, in which the patient is treated as a full-fledged person [1]. In turn, the universal theory of behaviour in interaction with dying people proposes four attitudes of behaviour: partner, hetero-centric, religious and indifferent [2,3]. The above theory confirms an existential–spiritual view, which ethically shows a man in all his existence. It should be added that the spiritual–internal activity of man is most often directed at the world of religious values. The spiritual view on death is a very interesting space in which the inner transcendental reflection of man on the purpose of his existence takes place. In contrast, the existential approach to death and relationships with dying allows you to construct a palette of many variables from which you can deal with this problem. However, the emotions accompanying patients during the experience of death can only be partially verbalized and shared with those who participate in this process. They are usually doctors and nurses, i.e., people practising professions of great social trust and great responsibility for human life. At the same time, these are two professions often on the verge of life and death. One can venture to say that they are so-called assistance professions that participate in a difficult mission of escorting from and saying goodbye to this world. The outlined perspective allows you to enter medical and nursing staff into the existential and spiritual space. It, in turn, allows one to notice the human attitude of the staff, expressed in competence, skills, knowledge and empathy and, on the other hand, a spiritual attitude marked by a mystery of crossing the death threshold. The above theory is part of the definition of attitude, which includes a fairly tight integration of the intellectual, emotional and volitional sphere of man and is shaped on the path of a person’s development and depends on the environment, acquired knowledge and experience [4,5]. Attitude is usually an established human behaviour associated with personality. It may change as a result of existential experience, especially the difficult situation on the border between life and death. Attitudes can also change due to interaction with the dying [6]. Therefore, the problem of the approach to death seems to be a very important element in the relationship between staff and the patient. Testing attitudes using a selected standardized tool, DAP-R-PL, is only one of the possibilities of describing such a difficult problem as the attitude of a person towards death. The collected data, by compiling the correlation of five individual factors and factor analysis, allow us to present a subjective assessment of medical and nursing personnel, taking into account the emotions and opinions of respondents, i.e., the psycho-existential state at a given moment. The participation in this study of doctors and nurses confirms their openness to the problem of death, which may contribute to positive interactions with the patients under their care. Research conducted in this space confirms that conversations with patients about the end of life can bring positive results [7]. However, analysing the literature on the attitudes of medical workers towards death, one can also see such opinions that speak of fears in interactions with terminally ill people, indicating that this problem is still open and requires new solutions. It should be emphasized that doctors and nurses are people who every day come into contact with people at the end of their lives and present various behaviours. It is noteworthy that medical and nursing personnel who come into contact with patients’ deaths also enter into interaction with dying families. That is why some specialists rightly emphasize that doctors and nurses should master the techniques of personal interaction well to meet the expectations of families who lose their loved ones [8]. The current time of the pandemic shows, in a specific form, the interactions of doctors and nurses with dying people and their relatives. High expectations are placed on the medical and nursing staff who find themselves in new operating conditions. At the same time, few people pay attention to the fact that staff who existentially come into contact with the death of their patients are in great psychological discomfort [9]. In addition, numerous studies indicate that some doctors, even after special training and courses on the approach to death, are not able to cope with this problem. The doctors’ opinions on the help offered are worrying because the educational tools used are not effective for maintaining correct interaction with the dying. Medics believe that encountering the death of their patients is a difficult experience, but it offers more benefits than the courses they took and training [10]. The spiritual attitude of doctors and nurses towards dying people plays an important role in the approach to this problem. It should be added that the spiritual attitude towards death is primarily determined by generated values from religion. Scientists dealing with the behaviour of doctors towards death prove that religion has a significant impact on ethical attitudes towards dying people. Respondents’ religious attitudes were measured using the DAP-R-PL (AA) questionnaire, the fourth factor of which includes questions about attitudes towards death. In the available literature, medical and nursing staff often refer to death as a transition, which may suggest that life continues after death. The above opinion indicates an ethical approach to death, but above all a spiritual attitude and a religious view of this phenomenon [11,12]. The results of other studies conducted in the medical world, e.g., among Palestinian and Polish medical students, confirm the thesis that people preparing to work in medical services have a positive existential and religious attitude to death [13,14]. This is also confirmed by the research conducted among students of Polish and English-speaking medical faculties, where Polish respondents show greater empathy towards death and the dying [15]. The purpose of the study is to evaluate the existential and spiritual attitudes of medical and nursing staff in the face of death.

## 2. Materials and Methods

In total, 220 people, i.e., 110 doctors and 110 nurses, participated in the study, aged between 22 and 50 years old. Respondents were recruited from 16 regions during medical courses and workshops. The study group was dominated by women, both in the group of medical (62.7%) and nursing (76.4%). The majority of respondents were under 30 years of age. Detailed data are presented in Table 1.

The study used a diagnostic survey method using a standardized research tool, i.e., the Questionnaire of Attitudes Against Death (DAP-R-PL) by Wong, Reker and Gesser, whose adaptation to Polish conditions was completed by Brudek and Sękowski [16]. This questionnaire consists of two parts. The first contains sociodemographic questions, while the second part of the questionnaire includes questions about the attitude of respondents to death. This questionnaire includes 32 questions/items (including 31 diagnostic) assessed by the respondent based on the 7-point Likert scale ranging from 1 (strongly disagree) to 7 (strongly agree). The questions/positions contained in the scale constitute five dimensions of attitudes towards death: 1. Fear of Death (FD); 2. Death Avoidance (DA); 3. Neutral Acceptance (NA); 4. Religious Acceptance of Death: Approach Acceptance (AA); 5. Escape Acceptance (AE). The range of collected results is in the range of 1–7 points. The higher the score, the more severe the attitude towards death. Statistical analysis was performed using the computer program R, version 3.5.0 and STATISTICA software.

In the paper, a two-step research procedure was applied. In the first, to present a structure of the analysed data, descriptive statistics were used, such as: number of valid cases, arithmetic mean, standard deviation, median, minimum, maximum. In the second one, the factor analysis was applied to a more profound study of the obtained results. The applied factor analysis makes it possible to discover the hidden dimensions of death as the phenomenon that is directly unobservable and group the answers of the respondents (their emotions and opinions), subjecting them to a broader interpretation. The structure indicator was also used and mathematical statistics were used, such as distribution matching tests, nonparametric correlations and differences significance tests. It was assumed that the probability *p* ≤ 0.05 means significant relationships and *p* ≤ 0.01 highly statistically significant. Based on the results of factor analysis, five variables were created based on the respondents’ answers, which arose after adding up the answers to individual questions. These variables included the respondents’ attitudes towards death.

1. Fear of Death (FD) related to questions about numbers 1, 2, 7, 18, 20, 21, 32, which contained the statements: death is undoubtedly an unpleasant experience; the prospect of my death raises anxiety in me; it worries me that death is inevitable; I am very afraid of death; I am terrified that death will mean the end of everything that I know; I am worried about the uncertainty about what will happen after death.
2. Death Avoidance (DA) included questions 3, 10, 12, 19, 26, with the statements: I avoid the thought of death at all costs; whenever a thought occurs in my head about death, I try to push it away; I try not to think about death; I completely avoid thinking about death; I try to have nothing to do with the subject of death.
3. Neutral Acceptance (NA) including questions 6, 14, 17, 24, 30 with statements: death should be seen as a natural, undeniable and unavoidable event; death is a natural aspect of life; I am not afraid of death but I am not waiting for death; death is part of life as a process; death is neither good nor bad.
4. Approach Acceptance (AA) with questions about numbers 4, 7,13, 15, 16, 22, 25, 27, 28, 31, where the statements were placed: I believe that I will be in heaven after death; death joins with entering the state of greatest satisfaction; I believe that heaven will be a much better place than this world; death is a union with God and eternal happiness; death brings the promise of a new and wonderful life; after death I expect to meet again with those whom I love; I see death as a transition to eternal and blessed place; death releases my soul; the only thing that gives me relief in the face of death is faith in life after death; I expect a new life after death.
5. Escape Acceptance (AE) with questions 5, 9, 11, 23, 29, including statements: death will end all my problems; death is an escape from this cruel world; death is delivery from pain and suffering; death is liberation from earthly suffering; I see death as a release from the burden of life.

The reliability of the scale was assessed using Cronbach’s alpha coefficient, which takes values from 0 to 1, where values above 0.7 mean the correct reliability of the scale. 

## 3. Results

The results obtained from the research using the Questionnaire About the Attitudes Against Death (DAP-R-PL) were verified in terms of scale reliability using Cronbach’s alpha coefficient. Its value for the whole scale, including 32 questions, was 0.865. For individual variables, it was as follows: Fear of Death 0.836, Death Avoidance 0.825, Neutral Acceptance 0.600, Approach Acceptance 0.903, Escape Acceptance 0.806. Analysis of individual dimensions of death showed that the examined staff obtained the highest average in the Approach Acceptance range (41.5 ± 15.0) and the lowest in the Death Avoidance dimension (17.7 ± 7.5). It has been observed that regardless of their profession, both medical and nursing staff are characterized by a religious attitude towards death, i.e., its acceptance results from the religious approach of the respondents. Other attitudes equally often presented by the respondents were Fear of Death (FD) and neutral attitude towards the analysed phenomenon (Neutral Acceptance NA) Table 2.

The conducted analysis showed moderate strength correlations between the examined variables. The strongest relationship was obtained between Fear of Death (FD) and Death Avoidance (DA) dimensions (*r* = 0.556; *p* < 0.05), and it is a positive relationship, i.e., personnel characterized by fear of death statistically significantly more frequently avoid thoughts and topics associated with death. It is also interesting that the attitude of Fear of Death is positively and moderately correlated with the attitude of Approach Acceptance (*r* = 0.366; *p* < 0.05) and negatively with Neutral Acceptance (*r* = −0.272; *p* < 0.05). There is also a moderate negative correlation between Death Avoidance and Neutral Acceptance variables (*r* = −0.302; *p* < 0.05) and a moderate positive correlation between Death Avoidance and Approach Acceptance (*r* = 0.298; *p* < 0.05). The Escape Acceptance variable is only associated with the Approach Acceptance variable (*r* = 0.380; *p* < 0.05). Detailed results are included in the Table 3. 

The Mann–Whitney U test was used to determine if there were statistically significant differences between the respondents’ attitudes towards death (FD, DA, NA, AA, AE).

**Figure 1:**
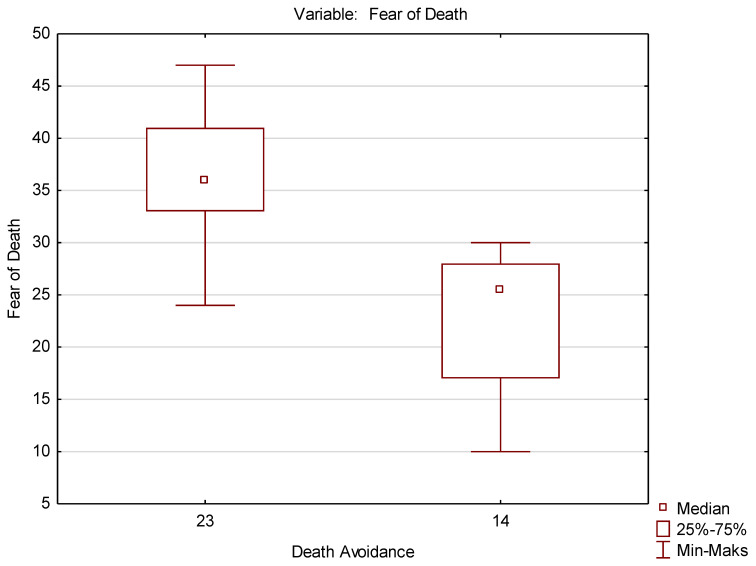
Box-whisker plot for FD and DA attitudes.

**Figure 2:**
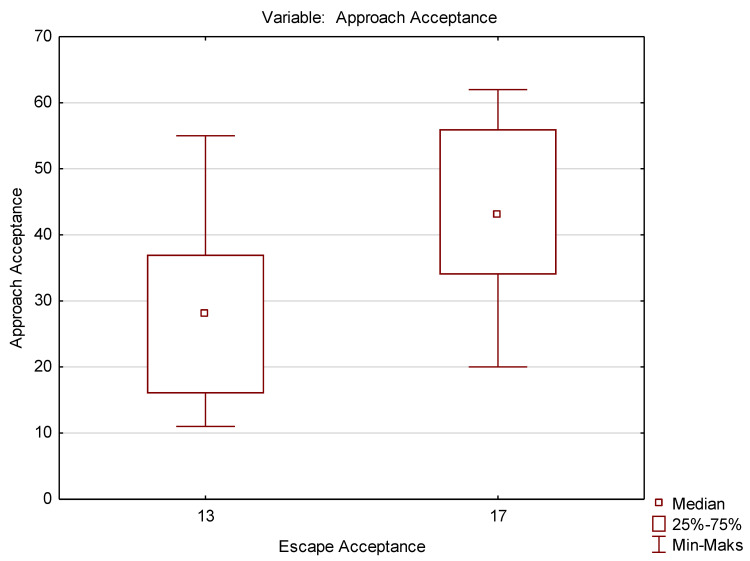
Box-whisker plot for AA and AE attitudes.

This test belongs to the group of non-parametric tests, used wherever the assumptions required for parametric tests (normal distribution, equality of variance) are not met. The above test was used to check the differences between all pairs of attitudes. Only the differences between the mean results obtained for the attitudes of FD and DA (*p* < 0.05) and AA and AE (*p* < 0.05) were statistically significant (Table 4 and Table 5). This is also confirmed by graphic interpretations: the box-whisker plot (Figure 1 and Figure 2).

Analysis of the results presented in Table 6 indicates the existence of moderate and high values of factor loadings of all items forming individual subscales, except for questions 1, 17, 30, which were rejected from the scale created because they were below 0.42. The values of factor loadings of the items making up the distinguished dimensions are: for Approach Acceptance, from 0.58 to 0.84; for Fear of Death, from 0.58 to 0.81; for Escape Acceptance, from 0.59 to 0.80; for Neutral Acceptance, from 0.60 to 0.84; for Death Avoidance, from 0.51 to 0.78. The results of factor analysis indicated a five-factor structure of the scale, which explains 58.42% of the variance. The first factor, explaining 19.18% of the variance, makes 10 theorems (4, 8, 13, 15, 16, 22, 25, 27, 28 and 31 theorems of the original version). The second factor, explaining 13.10% of the variance, brings together 6 theorems (2, 7, 18, 20, 21 and 32 theorems of the original version). The third factor explains 10.10% of the variance and brings together 5 theorems (5, 9, 11, 23 and 29 theorems of the original version). The fourth factor explains 7.07% of the variance and brings together 3 theorems (6, 14 and 24 theorems of the original version). The fifth factor explains 8.96% of variance and brings together 5 theorems (3, 10, 12, 19 and 26 theorems of the original version). The value of Cronbach’s *α* coefficient for the whole scale, including 32 theorems, is 0.87 (on a scale of 0–1). Cronbach’s *α* value for the Approach Acceptance factor is 0.93, for Fear of Death—0.85, for Escape Acceptance-0.81, for Neutral Acceptance—0.70 and for Death Avoidance—0.83.

## 4. Discussion

Issues regarding dying and death are difficult ethical topics which are undertaken by researchers with great restraint and caution. Although these issues are inseparable elements of human life and have been accompanying us since the beginning of human existence, we still feel the fear of them. This fear is caused by the reconciliation of these processes with the basic biological instincts of man. Images about death and attitudes can be very different, because they are marked by an ethical dimension and an individual approach to this problem. The doctors and nurses in their professional work often encounter the death of the patient and try to behave with calmness and self-control. However, positive and negative emotions always occur during such meetings. They are often dominated by disappointment, lack of hope or a sense of regret. The defence response of medical services is very often an attitude of fear, flight or avoidance of death. This confirms in this study the most visible attitude of Approach Acceptance, which comes first in the space of the other four attitudes. The required opinion, on the one hand, indicates the reflex of accepting death, and, on the other, it seems to be a temporary escape from a problem that we all sooner or later will face. An attitude of acceptance is often associated with the fear of death, which often accompanies this phenomenon. This is confirmed by studies conducted among medical and nursing personnel at the New York Cancer Hospital which show that respondents were the first to indicate the attitude of Fear of Death [17]. This behaviour can lead to adverse interactions with patients, because fear is most often the human body’s response to an external emergency. The presence is indicated by symptoms such as rapid heartbeat, increased secretion of adrenaline, trembling hands and voice. However, fear is a defensive reaction, thanks to which a person deals with anxiety, which the respondents cited and research indicated was in second place. Considering both attitudes, it should be noted that the basic function of fear and anxiety is signalling a threat, danger or conflict of motivation and, consequently, triggering appropriate responses to these situations, which, in the work of doctors and nurses, is an indispensable skill [18]. Our own research reveals the strongest relationship between two attitudes: Fear of Death and Death Avoidance, which determines the strength of mutual correlation. This behaviour may indicate a purely human approach to the subject of death, because man is naturally afraid of death and seeks a way of escape from it. 

The respondents’ opinion may also be a reflection of their daily interactions with the dying, which in turn shapes the attitude of fear. This behaviour confirms the thesis that medical and nursing services have an existential, ethical awareness of the importance of the phenomenon of death and do not treat it objectively, but see in this process people with their emotions. In other studies, it was found that low awareness of the problem of avoiding death and fear of it generates positive well-being [19], which in the case of medical and nursing staff can lead to unethical attitudes and indifference towards dying. Obtained results from the research conducted in the variable space: Fear of Death, Death Avoidance, Neutral Acceptance and Escape Acceptance, create an existential perspective and present the attitudes of medical and nursing staff towards the phenomenon of death. Respondents assessing the attitude of Fear of Death most often indicated the answer: the fact that death will mean the end of everything as I know it frightens me, which corresponds with another opinion, which says that the moment of death existentially closes everything a human has experienced and learned. It should be added that fear is an emotional reaction to an existential threat and is usually accompanied by a desire to escape, horror, sadness or anger. The most common answer to fear is an alternative to escape or fight. However, in the personal contact of doctors and nurses with the phenomenon of death, the fight to overcome fear is not easy. 

This is confirmed by the results of other studies conducted among representatives of medical and nursing staff indicating a correlation between the attitude of Fear of Death and Death Avoidance and Neutral Acceptance [20]. It is not easy to shape the ethical attitude of fighting this phenomenon. Therefore, an indicated alternative are workshops aimed at eliminating the fear of death and shaping positive ethical interactions with dying people [21]. Some scientists believe that fear of death may be milder when doctors and nurses maintain positive relationships with terminally ill people [22]. Nurses dealing with oncologically ill patients show a lower degree of fear of death, which is confirmed by the results of research on this phenomenon [23]. However, taking into account global solutions, the observation of specialists dealing with the fear of death (in selected geographical areas) showed that doctors and nurses solve this problem by applying to terminally ill entheogens to reduce their fear of death. However, researchers of this issue emphasize that these practices are not entirely safe and may in the future be an incentive for doctors and nurses to thus reduce personal fear of death [24].

The analysis of the conducted research indicates a moderate correlation relationship between the attitude of Fear of Death and the attitude of Approach Acceptance, which may suggest that respondents, fearing death, see in it the gate from which to be released from world problems, pain and suffering. The question arises whether the attitudes presented can translate into positive interactions with terminally ill people. The answer to this question may be the opinion obtained from similar studies, where it has been proven that almost 70% of doctors and nurses feel fear of talking to dying people [24]. Other reports generate an opinion that the existential fear of doctors and nurses from talking about death prevents their interaction with dying people [25]. This research shows that doctors and nurses believe that death is a natural aspect of life because they most often indicated this answer by describing their attitude in the Neutral Acceptance space, which, according to the results, is negatively correlated with Fear of Death. The above opinion corresponds to the results of research conducted among intensive care medical workers, where the Neutral Acceptance attitude was also dominant [26].

The results of this study also show the strength of linking the Approach Acceptance attitude with the Escape Acceptance attitude, where respondents most often chose the answer: I see death as a relief from the burden of this life. It is worth adding that the Escape Acceptance attitude is only correlated with Approach Acceptance, which is interesting from the existential–spiritual position. The first attitude through the terms contained indicates the release of the body from suffering, and the second containing spiritual determinants is the desired space to which man wants to go. The required opinion may generate the thesis that death is an escape from the hardships of everyday life, freeing man from physicality and the process of transition to spiritual space. Respondents describing their attitudes in this space (Approach Acceptance) most often marked the answer: I see death as a passage to an eternal and blessed place. It can be assumed that the opinion given presents the ethical approach of the respondents to the topic of death and, by presenting the spiritual attitude, indicates the axiology of personal religious life and openness to interaction with dying people.

## 5. Conclusions

The conducted research shows positive and ethical standards of attitudes of doctors and nurses towards death. An analysis of the five dimensions of death has shown that the presented opinions of both professional groups are convergent. The doctors and nurses present a mature accepting attitude towards death. This is due to religious beliefs as well as the ability to cope with the death of patients. Respondents presenting an attitude based on anxiety (Fear of Death) at the same time adopted the attitude of avoiding (Death Avoidance). In addition, the participants of the study combined the spiritual attitude (Escape Acceptance) only with the attitude of accepting death (Approach Acceptance). In the light of research, health care workers show great commitment to dying people in their existential and spiritual dimension.

## Figures and Tables

**Table 1:** Respondents by demographic characteristics.

	Staff Surveyed			
	**Medical**		**Nursing**	
	*n*	%	*n*	%
**Women**	69	62.7	84	76.4
**Men**	41	37.3	26	23.6
	**Age of Respondents**			
**Up to 30 years**	60	54.5	107	97.3
**Over 30 years**	50	45.5	3	3.7

**Table 2:** Selected descriptive statistics for individual death dimensions according to DAP-R-PL.

	Dimensions of Death				
	General Descriptive Statistics for Specific Dimensions of Death				
	Fear of Death	Death Avoidance	Neutral Acceptance	Approach Acceptance	Escape Acceptance
**M ± SD**	30.1 ± 9.8	17.7 ± 7.5	27.6 ± 5.2	41.5 ± 15.0	19.9 ± 7.7
**Me**	29.0	17.0	28.0	42.5	19.0
**Min–Max**	10.0–49.0	5.0–35.0	11.0–35.0	10.0–70.0	5.0–35.0
**Coefficient of Variation**	32.7	42.6	18.9	36.1	38.7
**Slant**	0.2	0.4	−0.6	−0.4	0.3
**Descriptive statistics for medical staff**					
**M ± SD**	**29.2 ± 10.2**	**17.1 ± 7.6**	**27.1 ± 5.8**	**42.8 ± 15.1**	**19.7 ± 7.7**
**Me**	28.5	16.0	28.0	44.0	20.0
**Min-Max**	10.0–49.0	5.0–35.0	11.0–35.0	10.0–70.0	5.0–35.0
**Descriptive statistics for nursing staff**					
**M ± SD**	**30.9 ± 9.4**	**18.3 ± 7.5**	**28.0 ± 4.5**	**40.1 ± 14.8**	**20.1 ± 7.8**
**Me**	30.5	18.0	28.0	41.0	19.0
**Min-Max**	13.0–49.0	5.0–35.0	17.0–35.0	11.0–66.0	5.0–35.0

M—average; SD—standard deviation; Me—median; Min—minimum; Max—maximum.

**Table 3:** Relationship between variables regarding attitudes to death.

		Dimensions of Death				
		Fear of Death	Death Avoidance	Neutral Acceptance	Approach Acceptance	Escape Acceptance
**Fear of Death**	*r*	1.000	0.556	−0.272	0.366	0.056
	*t*		9.887	−4.179	5.696	0.822
	*p*		0.05	0.05	0.05	0.412
**Death Avoidance**	*r*	0.556	1.000	−0.302	0.298	0.113
	*t*	9.887		−4.684	4.615	1.672
	*p*	0.05		0.05	0.000	0.096
**Neutral Acceptance**	*r*	−0.272	−0.302	1.000	−0.166	0.107
	*t*	−4.179	−4.684		−2.481	1.587
	*p*	0.05	0.05		0.014	0.114
**Approach Acceptance**	*r*	0.366	0.298	−0.166	1.000	0.380
	*t*	5.696	4.615	−2.481		6.060
	*p*	0.05	0.000	0.014		0.000
**Escape Acceptance**	*r*	0.056	0.113	0.107	0.380	1.000
	*t*	0.822	1.672	1.587	6.060	
	*p*	0.412	0.412	0.114	0.000	

*r*—Pearson correlation coefficient, *p*—significance level, *t*—student test.

**Table 4:** The results of the Mann–Whitney U test for variables: FD and AD.

Variable	Mann–Whitney U test (Continuity Corrected) (% of Variables) Relative to the Variable: Death Avoidance. The Results are Significant with *p* < 0.05000.								
	Sum. Rang Group 1	Sum. Rang Group 2	U	Z	*p*	Z Revised	*p*	N Group 1	N Group 2
**Fear of Death**	168.0000	63.00000	8.000000	3.274431	0.001059	3.276560	0.001051	11	10

**Table 5:** The results of the Mann–Whitney U test for variables: AA and AE.

Variable	Mann-Whitney U test (Continuity Corrected) (% of Variables) Relative to the Variable: Death Avoidance. The Results are Significant with *p* < 0.05000.								
	Sum. Rang Group 1	Sum. Rang Group 2	U	Z	*p*	Z Revised.	*p*	N Group 1	N Group 2
**Fear of Death**	65.5000	144.5000	20.5000	−2.16525	0.030369	−2.17015	0.029996	9	11

**Table 6:** Factor load matrix.

	Factor				
Question	1	2	3	4	5
**T4**	0.80				
**T8**	0.58				
**T13**	0.78				
**T15**	0.82				
**T16**	0.82				
**T22**	0.71				
**T25**	0.84				
**T27**	0.63				
**T28**	0.77				
**T31**	0.78				
**T1**		*			
**T2**		0.68			
**T7**		0.78			
**T17**		*			
**T18**		0.67			
**T20**		0.58			
**T21**		0.81			
**T32**		0.76			
**T5**			0.59		
**T9**			0.76		
**T11**			0.68		
**T23**			0.72		
**T29**			0.80		
**T6**				0.60	
**T14**				0.84	
**T24**				0.80	
**T3**					0.62
**T10**					0.66
**T12**					0.75
**T19**					0.78
**T26**					0.51
**T30**					*

* Source: own calculations × Factor loadings below 0.42 have been dropped. The method of extracting factors—the main components. Rotation method—Varimax standardized.

## Data Availability

Data are available upon reasonable request.
